# The Efficacy of Fosfomycin as Antibiotic Prophylaxis for Transrectal Prostate Biopsy and Impact on Lower Urinary Tract Symptom After Biopsy: A Prospective Study

**DOI:** 10.5152/tud.2023.23030

**Published:** 2023-07-01

**Authors:** Andreia Cardoso, Jorge Ribeiro, Rafael Araújo, João Pimentel Torres, Paulo Mota

**Affiliations:** 1 Department of Urology, Hospital de Braga, Braga, Portugal; 2 University of Minho, School of Medicine, Braga, Portugal

**Keywords:** Prostate, biopsy, complications, antibiotic prophylaxis, lower urinary tract symptoms, fosfomycin

## Abstract

**Objective:**

Due to fluoroquinolone resistances worldwide, valid alternatives for antibiotic prophylaxis for transrectal ultrasound-guided prostate biopsy are needed, thus, we aimed to evaluate the efficacy, safety, and tolerability of prophylactic fosfomycin versus other oral prolonged antibiotic regimens, in preventing complications after transrectal ultrasound-guided prostate biopsy.

**Methods:**

In this prospective study, patients submitted to transrectal ultrasound-guided prostate biopsy were divided into 2 groups according to the prophylactic antibiotic scheme performed: “short” (2 fosfomycin doses) versus “long” (antibiotic ≥ 8 days). One week and 1 month after transrectal ultrasound-guided prostate biopsy, we assessed complications’ occurrence (lower urinary tract symptoms, fever, sepsis, hemorrhage) and adverse drug reactions.

**Results:**

We included 244 patients (fosfomycin n = 178, “long” antibiotic n = 66). The only significant difference between groups was higher lower urinary tract symptom incidence 1 month after transrectal ultrasound-guided prostate biopsy in fosfomycin patients (16.85% vs. 6.06%, *P* = .031). However, after 1 week, lower urinary tract symptoms were tendentially frequenter on “long” antibiotic group (31.81% vs. 25.84%, *P* = .059). Infectious and hemorrhagic complications rate, adverse drug reactions, and recurrence to health services were similar between groups, and significantly decreased between the first week and first month.

**Conclusion:**

Antibiotic prophylaxis seems to impact lower urinary tract symptoms after transrectal ultrasound-guided prostate biopsy. Fosfomycin may provide slightly better outcome on the immediate period, while “long” antibiotic courses lead to significantly less lower urinary tract symptoms 1 month post-transrectal ultrasound-guided prostate biopsy, perhaps by preventing incipient prostatitis phenomena. Future directed studies should clarify these findings. Still, it seems feasible to ally fosfomycin advantages with noninferior safety, efficacy, and tolerability, allowing to reserve “long” regimens to other contexts. This is especially relevant in centers where transperineal biopsies are still not possible.

**Main Points**

- Fosfomycin is a feasible prophylactic antibiotic for transrectal prostate biopsies, presenting similar security, efficacy, and tolerability than full 8-day cycle of other oral antibiotic regimens.
- Fosfomycin seems to present better outcomes on the first week after biopsy, but slightly higher lower urinary tract symptoms incidence 1 month after biopsy, what may reflect a protective role of full 8-day antibiotic cycle on post-biopsy incipient prostatitis phenomena.
- Transrectal prostate biopsy is a well-tolerated procedure, with mild patient complaints mostly on the first week, which significantly resolve in 1 month.

## Introduction

Prostate cancer (PCa) is the second most prevalent, and the fifth main cause of death due cancer in men. Prostate cancer detection has been increasing worldwide, mainly due to large-scale use of the prostate-specific antigen as a screening tool for asymptomatic man.^1^

Prostate cancer definitive diagnosis requires histopathological confirmation, usually obtained through prostate biopsy (PB). The paradigm has recently changed, and, currently, the European Association of Urology highly recommends the transperineal route for PB, due to the lower risk of infectious complications, instead the transrectal ultrasound-guided prostate biopsy (TRUSPB).^2^ However, in some centers, TRUSPB remains the main technique for PB.

Prostate biopsy is usually well tolerated, with a low risk of major complications and rare mortality, but frequent minor complications, mostly hemorrhagic and temporary worsening of lower urinary tract symptoms (LUTS).^2,3^

Infection is a main concern, once it can present variable gravity, but can lead to sepsis and death.^2,3^ In TRUSPB, inoculation of rectal bacteria is the main infection mechanism.^4^ Antibiotic (Atb) prophylaxis has been shown to significantly reduce infectious complications, so it is globally recommended for TRUSPB, as well as rectal cleansing with povidone-iodine.^2,5^

Post-TRUSPB infections rate has raised in the last years, with the additional problem of growing bacterial Atb resistance. Fluoroquinolones (FQ) have been the gold-standard Atb prophylaxis for years, however due to high rate of bacterial resistance, FQ avoidance is now strongly recommended.^5-8^

Thus, alternative Atb schemes have been studied, comprising monotherapy, and targeted and augmented prophylaxis. Due to its implementation simplicity and efficacy, monotherapy with alternative Atb such as fosfomycin, cephalosporin, or aminoglycoside has been mainly used.^2,9^

Fosfomycin, in particular, is an interesting alternative to TRUSPB prophylaxis, once: it presents low rates of bacterial resistance, and no crossed resistance with Atb daily used^10^; its security profile is well known since it has been commonly used for decades; is easy to take; and its efficacy in preventing infectious complications compared to FQ has been shown in quality studies.^11,12^ Still, its use remains not widespread in the urologic community, that reveals some resistance to its wide and routine implementation, as shown by Dermitas et al.^13^

Thus, Atb prophylaxis previous to TRUSPB is a well-established and central need, in order to diminish associated infectious complications. However, the ideal drug and Atb schemes are yet to be defined. So, we aimed to assess the efficacy and tolerability of the promising short course with 2 oral doses of fosfomycin, with other oral “long” Atb regimens (≥8 days).

## Material and Methods

We designed a single center, descriptive, analytical, observational, and prospective (nonrandomized) study. It was approved by Hospital de Braga Ethics Committee on 02-12-2020, Ref.196_2020.

Our main objective was to evaluate the efficacy of oral fosfomycin prophylaxis (“short” scheme, 2 doses), compared to other oral “long” Atb course (≥8 days), in preventing post-TRUSPB complications. Secondarily, we aimed to compare the tolerability of both regimens. 

Eligible subjects were patients that met the following inclusion criteria: ≥18 years old, capable of giving informed consent or with a caregiver, submitted to TRUSPB on an outpatient basis, from October 2020 to October 2021, in our department, and that have performed oral Atb prophylaxis. The exclusion criterion was impossibility of completing study protocol. Written informed consent was obtained from all subjects before study inclusion.

Relevant baseline data collected were age, weight, height, calculated body mass index, urine culture (UC) result, and history of any Atb taken 3 months before TRUSPB. Urine culture was considered positive if there were >10^5^ colony forming units/mL of ≥1 bacterial strain.

Subjects were divided into 2 groups according to the oral Atb prophylactic scheme performed: “short” (fosfomycin 3000 mg, taken on the previous, and on the day of TRUSPB) versus “long” (Atb ≥ 8 days, started ≥1 day before TRUSPB). 

Then, we conducted 2 interviews (1 week and 1 month after TRUSPB), in which we applied 2 questionnaires to assess the occurrence of: post-biopsy complications (such as: dysuria, frequency, urgency, suprapubic discomfort, hemorrhage, and fever) and adverse drug reactions (ADR) specific for each Atb.^14-17^ We also asked patients if they have had any healthcare services need (HCSN) due to TRUSPB complications or Atb ADR.

For result analysis, post-TRUSPB complications were grouped as follows:

- Lower urinary tract symptom: dysuria, frequency, urgency, and suprapubic discomfort, in the absence of fever (body temperature ≥ 38ºC);
- Infectious complications: febrile urinary tract infection (UTI) (when LUTS are associated with fever in the absence of other infectious focus) and sepsis (according to the international consensus Sepsis-3)^18^;
- Hemorrhagic complications: hematuria, hematospermia, and rectal bleeding.

Naranjo et al algorithm^19^ was used to establish an eventual causality link between the suspicious ADR and the Atb taken.

Besides Atb prophylaxis, all patients applied a rectal enema (sodium citrate + sodium lauryl sulfoacetate, 5 mL, 450 mg/5 mL + 10 mL, or sodium docusate + sorbitol, 67.5 mL, 10 mg + 13400 mg) on the previous day, and a few hours before TRUSPB. Transrectal ultrasound-guided PB was performed by 1 of 3 urologists, with patient placed on left lateral decubitus, using a GE LOGIC S8®—General Ultrasound and a semiautomatic BIP-HistoCore® HC biopsy device (18G) with adjustable penetration depth tru-cut needle (18-25 mm), after peri-prostatic block with 10 mL of lidocaine 10 mg/mL. At least a 12-core systematic PB was always performed, with possible magnetic resonance imaging directed additional cores.

### Statistical Analysis

Data analysis was performed using Statistical Package for the Social Sciences version 26.0 (IBM SPSS Corp.; Armonk, NY, USA). A *P*-value <.05 was considered statistically significant.

Normality was assessed using Kolmogorov–Smirnov test, asymmetry, kurtosis, and visual assessment of histograms. Given the sample size (n > 100), parametric tests were used even if there was not a normal distribution, and results were compared with nonparametric tests. In case of normality, mean and SD were used, if not, median and interquartile range were used. For distribution comparison, for independent samples, Student’s *t*-test was used; otherwise, the Mann–Whitney test was used. Absolute and relative frequencies were presented. Comparison between groups and time frames were performed using Person’s chi-square (*X* ^2^), Fisher’s exact test, or McNemar test. The relevant *P*-values, effect sizes, d from Cohen, *t*-values, *X* ^2^ statistical value, and Phi (ɸ) were duly presented.

## Results

A total of 244 patients (fosfomycin n = 178, “long” Atb schemes n = 66) were included, after exclusion of 6 subjects due to impossibility to perform both scheduled assessments.

“Long” Atb group comprised: cefixime 400 mg id (n = 42), cefuroxime 500 mg bid (n = 13), ciprofloxacin 500 mg bid (n = 6), prulifloxacin 600 mg id (n = 3), amoxicillin/clavulanic acid 875 + 125mg bid (n = 1), and trimethoprim/sulfamethoxazole 160 mg/800 mg bid (n = 1).

Age varied between 43 and 90 years old. Table 1 presents detailed baseline characteristics. The only significant difference found was the higher frequency of positive UC in “long” Atb group (2.9% vs. 13.6%, *P* = .003).

Globally, about a third of the patients in both groups did not experience any complication in the first week after TRUSPB, whereas after 1 month, most of them did not report any complication (Table 2). There was a significative reduction in complications from the first week to the end of the first month, in both groups (*P* < .001, Table 3). Additionally, patients who underwent “long” Atb schemes showed significantly less complications on the first month, compared to fosfomycin group (16.7% vs. 32.0%, respectively, *P* = .017, Table 3). 

Next, we particularly analyze each subtype of complication.

There were statistically significant differences regarding LUTS, both temporally, and between groups at the end of first month (Table 3). Inside each group, LUTS frequency was significatively lower after 1 month (fosfomycin 16.85% vs. 25.84% at first week, *P* = .010; and “long” Atb 6.06% vs. 31.08% at first week, *P* < .001). Besides, fosfomycin patients presented significatively more LUTS at first month after TRUSPB, than “long” Atb subjects (16.85% vs. 6.06%, respectively, *P* = .031).

Hemorrhagic complications were the most frequent complications in both groups. Hematuria was the most prevalent symptom in the first week, referred by around half of the patients in both groups, followed by hematospermia (Table 2). One month after TRUSPB, hematuria remained the most reported complaint in fosfomycin group (14.61%), while in the “long” Atb patients, it was hematospermia (7.58%). No significant differences between groups on bleeding complications rate were found at any timeframe. Still, there was a significant decrease (*P* < .001), from the first week to the first month, within each group (Table 3).

All the reported infectious complications occurred in the first week post-TRUSPB, without significant differences between groups (Table 3). There was a total of 5 febrile UTIs (fosfomycin n = 3, 1.69%; “long” Atb n = 2, 3.03%) and 1 sepsis (on “long” Atb group, 1.52%) (Table 2).

There was no significant association between complications and the Atb intake in the 3 months prior to TRUSPB or the UC result, at any time, neither any group (Table 4).

Adverse drug reactions were similar in both groups, in both time assessments (first week around 18.0%, first month 3.0%-4.5%), with a significant decrease in ADR frequency, from the first week to the end of the first month (Table 3).

During the first week, 17 patients revealed HCSN, while at 1 month it was 9 patients, without significant differences between groups, neither at any time point (Table 3). The only case requiring hospital admission was the sepsis.

## Discussion

Prostate biopsy is one of the urologic procedures more frequently performed in civilized countries, in line with the high incidence of PCa. Although generally well tolerated, PB presents frequent minor complications (mainly hemorrhage and LUTS), but also a potential for major complications, though rare, that consist mainly in infection.^2,3^

The benefit of Atb prophylaxis in the reduction of infectious complications post-TRUSPB, HCSN, and hospital admission is well-established. So, its routine use is widely recommended, and even evidence-based in a systematic review and meta-analysis.^2,5^ Nowadays, the main question is what is the best prophylactic regimen for these patients. Fluoroquinolones have been the gold standard, but due to the high prevalence of bacterial resistance, mainly *E. coli*, FQ use is now discouraged (also evidence based in a systematic review and meta-analysis),^5,6^ and alternatives have been sought.

Our main goal was to demonstrate, prospectively, that 2 doses of oral fosfomycin, a short prophylactic scheme, may be safe, effective, tolerable, and noninferior in preventing post-TRUSPB complications, even when compared with therapeutic Atb course (≥8 days).

Regarding baseline data, the only difference found was that patients prescribed with “long” Atb presented significantly more positive UC previous to TRUSPB (13.6% vs. 2.9%, *P* = .003, Table 1). This may reflect a selection bias by the urologist when prescribing the prophylaxis,^13^ assuming that bacteriuria, even if asymptomatic, would deserve directed and therapeutic Atb course, given the performance of an invasive procedure as TRUSPB. The absence of TRUSPB histopathological results may be a limitation, however its correlation with biopsy complications can also be dubious and not straightforward. 

Lista et al^20^ reported a similar rate of complications 1 month after TRUSPB (22.6%, *P* = .17) with prophylactic fosfomycin or ciprofloxacin. Contrariwise, we found a significantly higher complication rate at 1 month with fosfomycin (Table 2, 32.0% vs. 16.7% “long” Atb, respectively, *P* = .017). This may be due to the discrepancy in the number of patients in each group (fosfomycin n = 178, “long” Atb n = 66), however, a long-term protection from “long” Atb cannot be excluded. 

It is estimated that 6%-25% of patients report new or aggravated LUTS post-TRUSP, especially the ones with larger prostates,^21^ an element that we did not consider, being a limitation of our study. Another limitation is the nonuse of an objective questionnaire as IPSS to assess LUTS pre- and post-biopsy, which would have strengthened the significance of our findings. Lower urinary tract symptoms frequency was similar between groups in the first week, slightly superior in “long” Atb group (Table 2). However, 1 month after TRUSPB, not only LUTS rate was significantly lower in each group comparing with first week, but also LUTS reduction was greater in “long” Atb patients, that finished with a LUTS rate statistically inferior to fosfomycin group (Table 3, 6.06% vs. 16.86%, respectively, *P* = .031). This may be due to the different group patients’ number, however this may reflect a beneficial effect of long Atb regimens, perhaps by preventing the post-PB incipient prostatitis phenomena.

A limitation on our analysis of LUTS is the absence of data about the previous use (or not) of alpha blocker or anticholinergic in each group. We did not specifically evaluate it, however, since the intake of these medications was not altered due to TRUSPB, we believe this might be a minor limitation. 

Hemorrhagic complications were the most frequent, which is in line with other studies, which reported the following: hematuria 10%-84%, hematospermia 1.1%-92.6%, rectal bleeding 1.3%-45%.^3^ We found hematuria to be the commonest complication in both groups, especially in the first week (Table 2), while hematospermia rate was 19.66% vs. 15.15%, and rectal bleeding 8.43% vs. 19.66%, in the first week, for fosfomycin versus “long” Atb, respectively (Table 3). Overall, hematospermia is known to be one of the most inconsistent variables among studies, which may reflect cultural stigmas, and issues related to the age and sexual life of patients, so its frequency may also be underestimated in our study. We did not evaluate the use of antiplatelet/anticoagulant agents, which may be a limitation. However, in our center, every patient equally suspends the intake of these medication prebiopsy, thus reducing this possible confounder bias (only acetylsalicylic acid is maintained in high-risk patients).

Infectious complications post-TRUSPPB are estimated to occur in 5%-7%, with need for hospital readmission in 1%-3%, sepsis in 0.1%-5.0%, and mortality in 0.1%-1.3% patients.^11,22^ Some studies reported a clear advantage of fosfomycin compared with FQ (febrile UTI 0.5%-0.9% vs. 3.4%-5.2%, and sepsis 0.3% vs. 1.8%, respectively).^10,23^ We also found a lower rate of febrile UTI in fosfomycin group (1.69% vs. 3.03%, Table 2), and only 1 case of sepsis (1.52%), in a patient that have taken ciprofloxacin and presented a resistant *E. coli* on UC, which was also the only needed hospital readmission (0.4%). Thus, we highlight that the reduced number of this events prevents us to draw definite conclusions. However, even using fosfomycin in a considerable number of patients, there were no infections reported after the first week post-TRUSPB, so we must truly avoid FQ as recommended, in order to avoid complications for our patients, and even to prevent the development of greater bacterial resistances.

In accordance with a previous systematic review and meta-analysis,^24^ we verified no association between complications and either UC result, neither Atb taken in the previous 3 months (Table 4). Only the intake of FQ in the past 6 months has been associated with a higher risk for colonization with resistant *E. coli* ^7^; however, FQ should be avoided, and thus, we also agree that routine UC previous to TRUSPB in asymptomatic man is not cost-effective, and, as reported, asymptomatic bacteriuria treatment pre-TRUSPB has no significant benefit for infection risk and might contribute to bacterial resistance development.^25^

Adverse drug reactions were not a relevant issue, and fosfomycin tolerability was similar to other Atb (Table 3), as reported previously in a systematic review and meta-analysis.^24^

Globally, there was a significant reduction in complications and ADR from the first week to the end of the first month, without significant differences between groups (Table 3), as expected.^23,26^ This is in line with the only available study, to the best of our knowledge, in which also 2 timeframe assessments were made, with significant symptoms improvement in between.^27^ We consider this justifies, consequently, and though without statistical significance, the roughly half reduction in HCSN along the first month (Table 3). 

Summarizing, the only significant unfavorable result with fosfomycin, comparing with “long” Atb, was LUTS prevalence 1 month after TRUSPB. Nevertheless, according to patients, these were light symptoms, self-limited, without impact on daily activities or HCSN. All other post-TRUSPB complications and ADR were similar between groups. 

Hence, fosfomycin presents as a good alternative to FQ and other “long” Atb, being equally effective, tolerable, and safe. Therefore, fosfomycin use may be preferred considering the other arguments in its favor: high efficacy against multidrug-resistant gram-negative bacteria (the most prevalent in rectal/urinary flora and the main agent of post-PB infections), favorable pharmacokinetic parameters, including a high prostatic penetration,^28^ and a low rate of resistant *E. coli* strains (<3%).^29^

Our study is not exempt of limitations: it was not randomized, so there is a possible bias in Atb selection from the patients’ urologist^13^; there is a discrepancy in patients’ number in each group (fosfomycin n = 178, “long” Atb n = 66), which may impact results’ analysis; some possible relevant variables, known to be associated with post-TRUSPB UTI,^4^ were not considered, such as, diabetes, prostate size, and number of PB cores retrieved, besides others (i.e., immunocompromise, suspicion lesions location); and we did not follow some of the recommendations^2^ such as rectal cleansing with povidone-iodine, the scheduled time for fosfomycin intake, or a true prophylaxis with 3-day Atb course, but rather performing a therapeutic scheme ≥8 days.

Despite its limitations, our prospective study addresses a theme highly relevant, not only on urologic field, but also in global medicine, once it approaches bacterial resistances and drugs used in daily practice for every doctors. With the recommendation for preferable transperineal route for PB, we could believe TRUSPB issues to be relevant no more. However, due to practical limitations in transperineal PB implementations in daily urology, it is still not a reality in several countries. So, we must improve TRUSPB results once it is the most (or only) available technique for many patients.

As final remarks, we highlight that TRUSPB is a well-tolerated procedure, causing some nondisturbing symptoms, mostly in the first week, but not leading to HCSN by patients. Fosfomycin appears to be noninferior preventing complications post-TRUSPB, while being equally tolerated, even in patients with previous positive UC or Atb intake in the last 3 months, when compared to oral “long” Atb regimens (≥8 days). Thus, it seems possible to ally fosfomycin advantages (few resistances, short scheme, easy intake) with its similar security, efficacy, and tolerability, enabling “long” Atb schemes to be reserved for clinical scenarios in which they are truly indispensable. This knowledge is of crucial relevance, especially in centers where transperineal PB is still not available, and TRUSPB remains the only possible route. 

However, further studies are suggested, preferably with larger samples, a multicenter, randomized controlled and prospective design, focusing on post-PB LUTS incidence and its impact on patients’ quality of life, and the possible association with different prophylactic Atb regimens, in order to confirm the safety and mid/long-term efficacy of prophylactic fosfomycin, and to achieve the best possible outcomes for men worldwide. 

## Figures and Tables

**Table 1.** Demographic and Clinical Data of Patients on Each Study Group

	Fosfomycin	Long Atb	*P*-Value	Effect Size	Statistical Test
Age (years), M ± SD	68.84 ± 9.17	69.05 ± 7.92	.874	*d *= ·-0.026	*t* = -0.159
BMI (kg/m^2^), Mdn/IQR	26.50 ± 4.38	25.89 ± 4.53	.746	*d* = -0.041	*t *= -0.325
Atb ≤ 3 months, (n = 36) n (%)	23 (12.9)	13 (19.7)	.185	*Φ* = 0.850	*X* ^2^ = 1.757
Positive UC, (n = 14) n (%)	5 (2.9)	9 (13.6)	**.003**	*Φ* = 0.207	FET

Atb, antibiotic; BMI, body mass index; d, *d* from Cohen; FET, Fisher’s exact test; IQR, interquartile range; M, mean; Mdn, median; n, number of patients; UC, urine culture; *Χ*², Pearson’s chi-square, value,* ɸ*, Phi.

**Table 2.** Complications, 1 Week and 1 Month After TRUSPB, According to the Oral Atb Prophylactic Regimen Taken

			Complications, n (%)								
			LUTS				Infection		Hemorrhage		
		None n (%)	Dysuria	Frequency	Urgency	Suprapubic discomfort	Febrile UTI	Sepsis	Hematuria	Rectal bleeding	Hemato-spermia
1 week	Fosfomycin n = 178	62 (34.83)	17 (9.55)	15 (8.43)	7 (3.93)	21 (11.80)	3 (1.69)	0	95 (53.37)	15 (8.43)	35 (19.66)
	Long Atb n = 66	23 (34.85)	9 (13.64)	9 (13.64)	1 (1.52)	8 (12.12)	2 (3.03)	1 (1.52)	36 (54.55)	7 (10.61)	10 (15.15)
1 month	Fosfomycin n = 178	121 (68.98)	10 (5.62)	8 (4.49)	7 (3.93)	15 (8.43)	0	0	26 (14.61)	1 (0.56)	12 (6.74)
	Long Atb n = 66	55 (83.33)	0	3 (4.55)	0	1 (1.52)	0	0	4 (6.06)	0	5 (7.58)

Atb, antibiotic; LUTS, lower urinary tract symptoms; UTI, urinary tract infection.

**Table 3.** Comparative Analysis of: Complications, ADR, and HCSN, 1 Week, and 1 Month After TRUSPB, According to the Oral Atb Prophylactic Regimen Used

n = 244	Fosfomycin	Long Atb	*P*	Effect-Size	Statistical Rest
	n = 178	n = 66			
Complications:					
First week	116 (65.2%)	43 (65.2%)	*P* = .998	*Φ* = 0.001	*X* ^2^ = 0.001
First month	57 (32.0%)	11 (16.7%)	****P** = .017**	*Φ* = -0.152	*X* ^2^ = 5.648
	*McNemar test*				
	***P*** ** < .001**	***P*** ** < .001**			
LUTS:					
First week	46 (25.84%)	21 (31.81%)	*P* = 0.059	*Φ* = 0.015	*X* ^2^ = 0.863
First month	30 (16.85%)	4 (6.06%)	***P*** ** = .031**	*Φ* = -0.138	*X* ^2^ = 4.677
	*McNemar test*				
	***P*** ** = .010**	***P*** ** < .001**			
Hemorrhagic complications					
First week	111 (62.4%)	38 (57.6%)	*P*=0.496	*Φ* = -0.044	*X* ^2^ = 0.463
First month	33 (18.5%)	7 (10.6%)	*P* = .137	*Φ* = -0.095	*X* ^2^=2.211
	*McNemar test*				
	***P*** ** < .001**	***P*** ** < .001**			
Infectious complications^†^ First week	3 (1.7%)	3 (4.5%)	*P* = 0.200	*Φ* = .082	*X* ^2^ = 1.642
				
ADR:					
First week	32 (18.0%)	12 (18.2%)	*P* = .971	*Φ* = 0.002	*X* ^2^ = 0.001
First month	8 (4.5%)	2 (3.0%)	*P* = 1.000	*Φ* = -0.033	FET
	*McNemar test*				
	***P*** ** < .001**	***P*** ** = 0.013**			
HCSN					
First week	13 (73%)	4 (6.1%)	*P* = 1.000	*Φ* = -0.022	FET
First month	7 (3.9%)	2 (30%)	*P* = 1.000	*Φ* = -0.021	FET
	*McNemar test*				
	*P* = .238	*P* = .687			

ADR, adverse drug reaction; Atb, antibiotic; FET, Fisher’s exact test; HCSN, healthcare services need; LUTS, lower urinary tract symptoms; n, number of patients; *Χ*², Chi-square value;* ɸ*, Phi.

^†^There were no reported infectious complications in either group, 1 month after biopsy.

**Table 4.** Comparison of Complications Attending to: Atb Intake in the 3 Months Prior to TRUSPB, and UC result

	Atb ≤ 3 months		*P*-value	Effect-Size	Statistical Test
	n (%)				
	No (n = 208)	Yes (n = 36)			
Complications:					
First week	135 (64.9%)	24 (66.7%)	*P* = 838	*Φ* = 0.001	*X* ^2^ = 0.042
First month	57 (27.4%)	11 (30.6%)	*P *= .697	*Φ* = 0.025	*X* ^2^ = 0.152

	**UC **n (%)		*P*-values	Effect-Size	Statistical Test
	Negative (n = 230)	Positive (n = 14)			
Complications:					
First week	152 (66.1%)	7 (50.0%)	*P* = .253	*Φ*=-0.079	FET
First month	67 (29.1%)	1 (7.1%)	*P* = .121	*Φ*=-0.114	FET

Atb, antibiotic; FET, Fisher’s exact test; n, number of patients; *P*, P-value; UC, urine culture; *Χ*², Chi-square value; ɸ, Phi.
